# Insights into the genomic homogeneity of Moroccan indigenous sheep breeds though the lens of runs of homozygosity

**DOI:** 10.1038/s41598-024-67558-w

**Published:** 2024-07-17

**Authors:** Szilvia Kusza, Bouabid Badaoui, George Wanjala

**Affiliations:** 1 Faculty of Agricultural and Food Sciences and Environmental Management, Centre for Agricultural Genomics and Biotechnology, University of Debrecen, Egyetem tér 1., 4032 Debrecen, Hungary; 2 Faculty of Sciences, Centre de Biotechnologies Végétales et Microbiennes, Biodiversité et Environnement, Mohammed V University in Rabat, Rabat, Morocco; 3 African Sustainable Agriculture Research Institute (ASARI),, Mohammed VI Polytechnic University (UM6P), Laâyoune, Morocco; 4 Doctoral School of Animal Science, University of Debrecen, Böszörményi út 138., 4032 Debrecen, Hungary; 5 Institute of Animal Sciences and Wildlife Management, University of Szeged, Andrássy út 15., 6800 Hódmezővásárhely, Hungary

**Keywords:** Genomic homogeneity, Indigenous sheep, Morocco, ROH, Genetics, Inbreeding

## Abstract

Numerous studies have indicated that Morocco’s indigenous sheep breeds are genetically homogenous, posing a risk to their survival in the challenging harsh climate conditions where they predominantly inhabit. To understand the genetic behind genetic homogeneity through the lens of runs of homozygosity (ROH), we analyzed the whole genome sequences of five indigenous sheep breeds (Beni Guil, Ouled Djellal, D’man, Sardi, Timahdite and Admixed).The results from principal component, admixture, Fst, and neighbour joining tree analyses consistently showed a homogenous genetic structure. This structure was characterized by an average length of 1.83 Mb for runs of homozygosity (ROH) segments, with a limited number of long ROH segments (24–48 Mb and > 48 Mb). The most common ROH segments were those ranging from 1–6 Mb. The most significant regions of homozygosity (ROH Islands) were mostly observed in two chromosomes, namely Chr1 and Chr5. Specifically, ROH Islands were exclusively discovered in the Ouled Djellal breed on Chr1, whereas Chr5 exhibited ROH Islands in all breeds. The analysis of ROH Island and iHS technique was employed to detect signatures of selection on Chr1 and Chr5. The results indicate that Chr5 had a high level of homogeneity, with the same genes being discovered across all breeds. In contrast, Chr1 displays some genetic variances between breeds. Genes identified on Chr5 included *SLC39A1, IL23A, CAST, IL5, IL13*, and *IL4* which are responsible for immune response while genes identified on Chr1 include *SOD1, SLAMF9, RTP4, CLDN1,* and *PRKAA2.* ROH segment profile and effective population sizes patterns suggests that the genetic uniformity of studied breeds is the outcome of events that transpired between 250 and 300 generations ago. This research not only contributes to the understanding of ROH distribution across breeds but helps design and implement native sheep breeding and conservation strategies in Morocco. Future research, incorporating a broader sample size and utilizing the pangenome for reference, is recommended to further elucidate these breeds’ genomic landscapes and adaptive mechanisms.

## Introduction

The escalating threat of climate change poses a substantial risk to biodiversity, manifesting in the alarming rate of species extinction. This phenomenon severely impacts global food security as the planet grapples with increasing temperatures and an increasing human population. The impending reality is stark: by 2030, global temperatures are projected to rise by 2 degrees Celsius, with a subsequent increment of 0.15 degrees Celsius every decade^1^. Such shifts in climate are expected to exert profound heat stress on livestock, significantly undermining agricultural productivity. The situation is further exacerbated by climate-induced health hazards that compromise animal immunity, necessitating urgent and sustainable interventions to preserve livestock productivity against the backdrop of climate change^2,3^.

Among small ruminants, sheep emerge as pivotal contributors to global animal protein sources. Their domestication in the Fertile Crescent around 11,000 years ago ushered in a series of evolutionary adaptations, enabling their proliferation across diverse geographical landscapes^4–6^. This evolutionary journey, shaped by both natural and selective breeding, has culminated in the emergence of distinct sheep breeds, each exhibiting unique phenotypic traits indicative of their adaptive capacities. Historically, sheep populations in North Africa have been in existence for seven millennia now, and they constitute a highly diverse sheep populations that have been bred under conventional agricultural practices for many years. Based on the historical records of African sheep, it is likely that the first sheep migrated from the center of domestication to Africa, possibly in response to droughts and other climate-related calamities^7^. Subsequently, there were many instances of dispersal, including a movement towards the North to Libya about 6700 BP, a movement towards the south to the middle Nile Valley, and a movement towards the west to the central Sahara around 6000 BP. The most recent dispersal episode occurred around 3700 years ago, towards West Africa according to MacDonald^8^. The evolution of sheep breeds in North Africa developed through many stages, resulting in the creation of sheep genetic resources that have historically adapted to local conditions. Nevertheless, as a result of ambiguous circumstances, the breeds have recently undergone intermixing and genetic homogeneity, resulting in the emergence of crossbred populations.Recent statistics highlight that, global sheep population is at over 1.28 billion, with Africa and Morocco contributing 10.3% and 1.75%, respectively^9,10^. The sheep demographic in Morocco predominantly features indigenous breeds, characterized by their resilience to the arid conditions prevalent in desert regions^11,12^. This resilience underscores the importance of understanding and harnessing their genetic adaptability, especially in the face of climatic adversities.

The advent of advanced genomic and bioinformatics tools has significantly enriched our comprehension of the genetic landscape, offering insights into the adaptability and diversity of animal genetic resources, particularly within Morocco^11,13–16^. While recent studies into the genetic diversity of Moroccan sheep breeds have revealed a general trend of between-breed genetic homogeneity, they concurrently highlight a substantial within-breed genetic diversity^13,17,18^. Moreover, the exploration of genomic signatures indicative of local adaptation has revealed unique genetic regions pivotal for environmental resilience, thereby accentuating the remarkable adaptive traits of these indigenous breeds^11^.

Despite the insights gained, the genomic underpinnings of homogeneity and adaptability, particularly through the lens of runs of homozygosity (ROH), remain under-explored. ROH, long homozygous regions traceable to a common ancestor, serve as crucial markers for assessing genome-wide inbreeding (Froh) and elucidating the genetic basis of homogeneity within the Moroccan sheep population^19^. This study, therefore, aims to dissect the ROH patterns across five Moroccan sheep breeds (Beni Guil, Ouled Djellal, D’man, Sardi, Timahdite and Admixed population) to unravel the historical events leading to their current homogenous genetic makeup. The “Admixed” sheep, refers to the local sheep population, which does not conform to the established phenotypes of known native breeds, suggesting these animals are likely hybrids derived from multiple genetic lineages.

## Results

### Population structure

**Figure 1:**
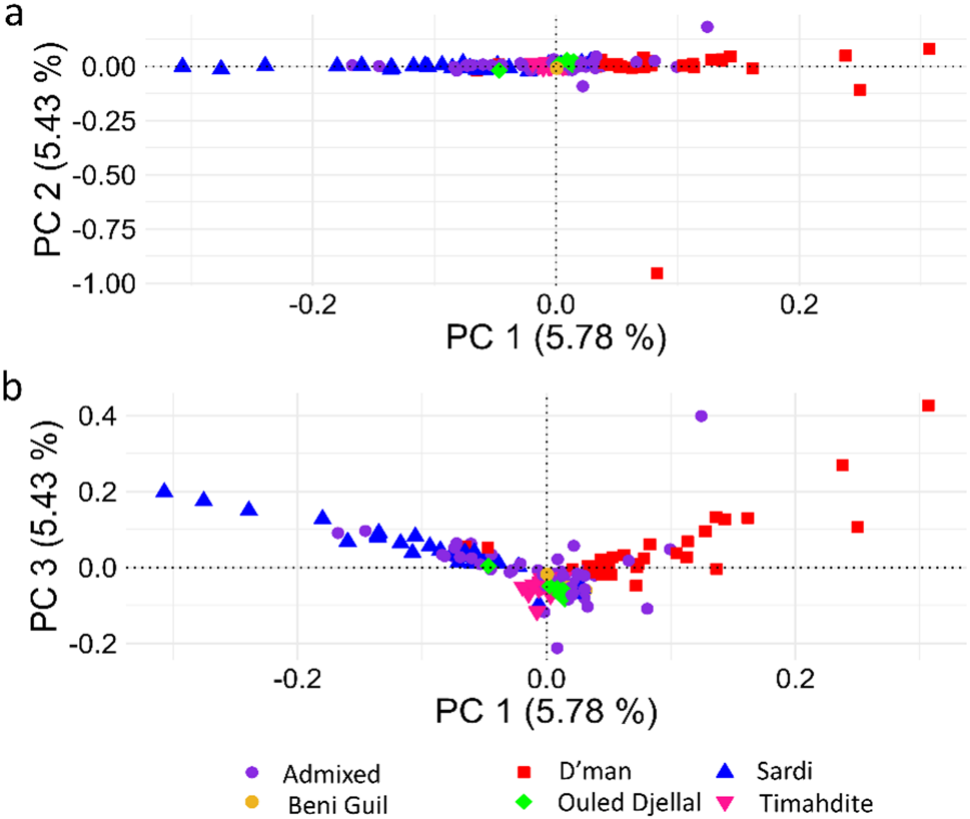
Population structure of six sheep breeds from Morocco as defined by principal component analysis. (**a**) PC1 versus PC2; (**b**) PC1 versus PC3.

As expected, the analysis of the first two principal components (PC1 and PC2) revealed no clear separation among the breeds. In total, PC1 and PC2 (Fig. 1a) accounted for 11.18% of the total variance observed among the populations, resulting in the breeds merging into a single notable cluster along PC1. The configuration of PC1 and PC3 (Fig. 1b) formed a “V” shape, mirroring the global sheep population structure previously outlined by Kijas et al.^20^. This observed pattern, however, did not serve to distinguish between the sheep breeds being studied. The wide spread of Admixed population indicates high level of within-breed genetic heterogeneity than other breeds.

**Figure 2:**
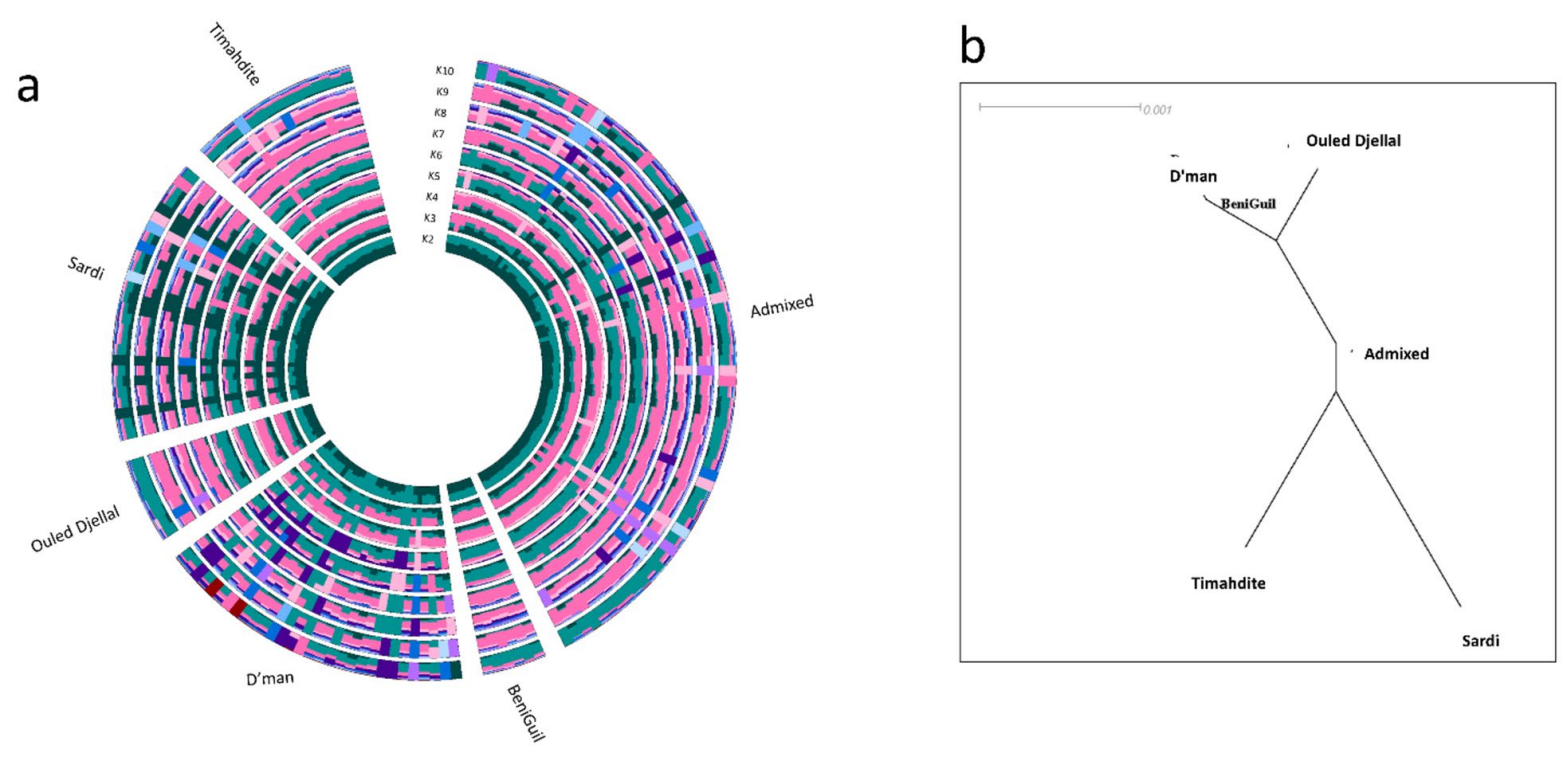
Population structure and relationship between breeds. (**a**): A circulat barchat showing ancestral member coefficients of each individual; (**b**): Neighbour joining tree showing relationship between breeds.

Figure 2a depicts the admixture software in a circular style. The cross-validation error achieved its minimal value at K = 2 (Supplementary Fig. S1). Like Fig. 1, there was no noticeable grouping or distinction across breeds. At a value of K = 2, there are two distinct clusters that are easily noticeable, but there is no one breed that looks to be uniform or homogenous. Remarkably, the admixture pattern remained consistent among all breeds, suggesting a significant degree of genetic similarity among the breeds being examined. As the value of K increased from 2 to 10, there was a proportional rise in the levels of mixing. Nevertheless, it was noted that there was a lack of homogenous breeds among the studied breeds across all K clusters. Figure 2b shows two distinct clusters of closely related breeds, agreeing with cross-validation results of K = 2. The first cluster, consisting of D’man, Ouled Djellal, and Beni Guil, exhibits a lower level of differentiation among its members, but is considerably dissimilar from the second cluster, which contains Timahdite and Sardi. The Admixed breed, as anticipated, is positioned at the center of the neighbor joining tree, indicating that it is highly admixed compared to all other breeds. These results can be interpreted alongside the FST (Supplementary Table 1). The highest pairwise FST was observed between Sardi and Dman (0.003, *p* < 0.001), while the lowest pairwise FST was observed between Ouled Djellal and Beni Guil (*P* -0.000, *P* > 0,05).

### Runs of homozygosity and inbreeding coefficients

**Figure 3:**
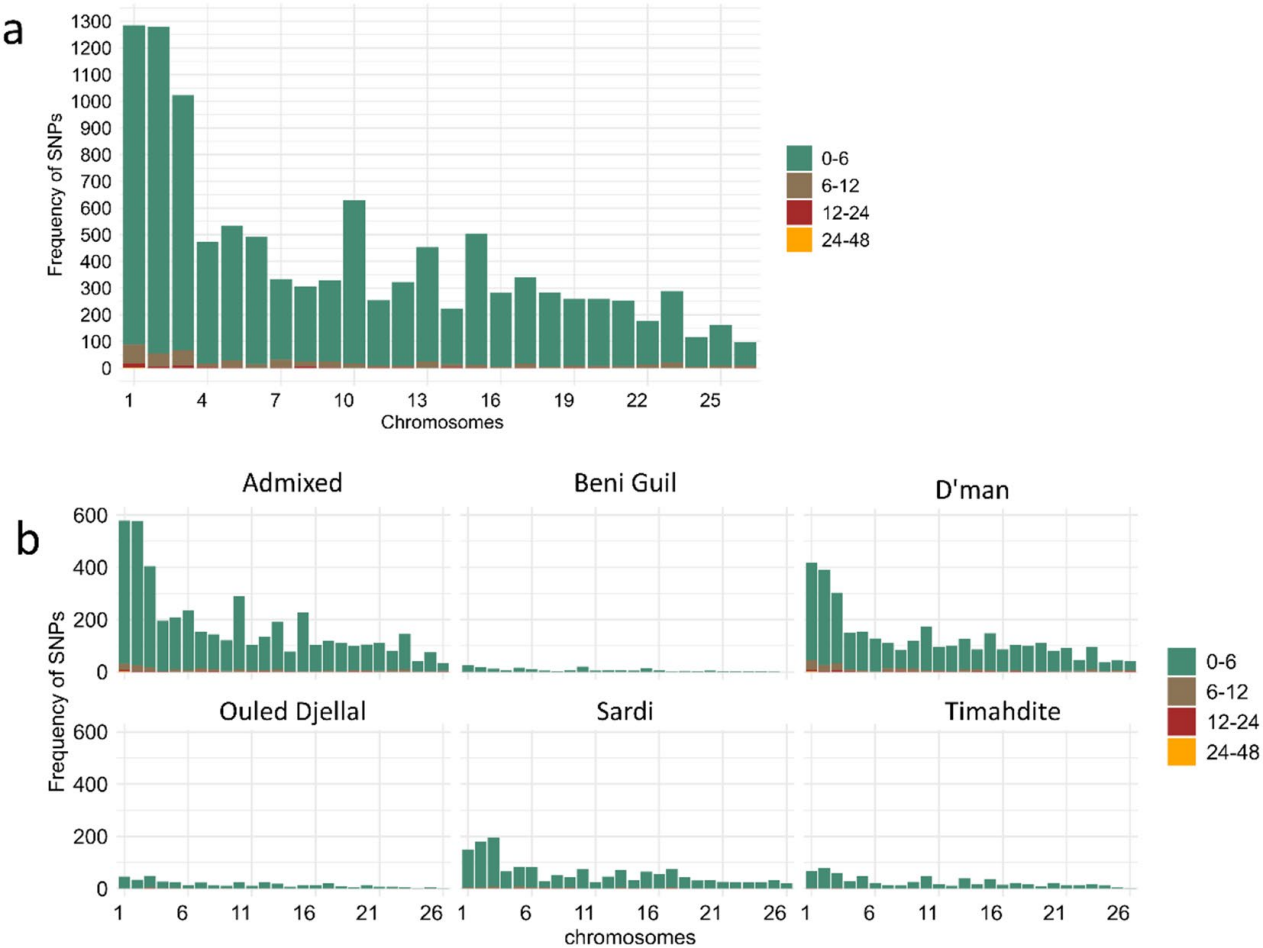
Number of SNPs in runs per chromosome. (**a**): Cumulative frequency for all breeds; (**b**): Frequency per breed.

**Figure 4:**
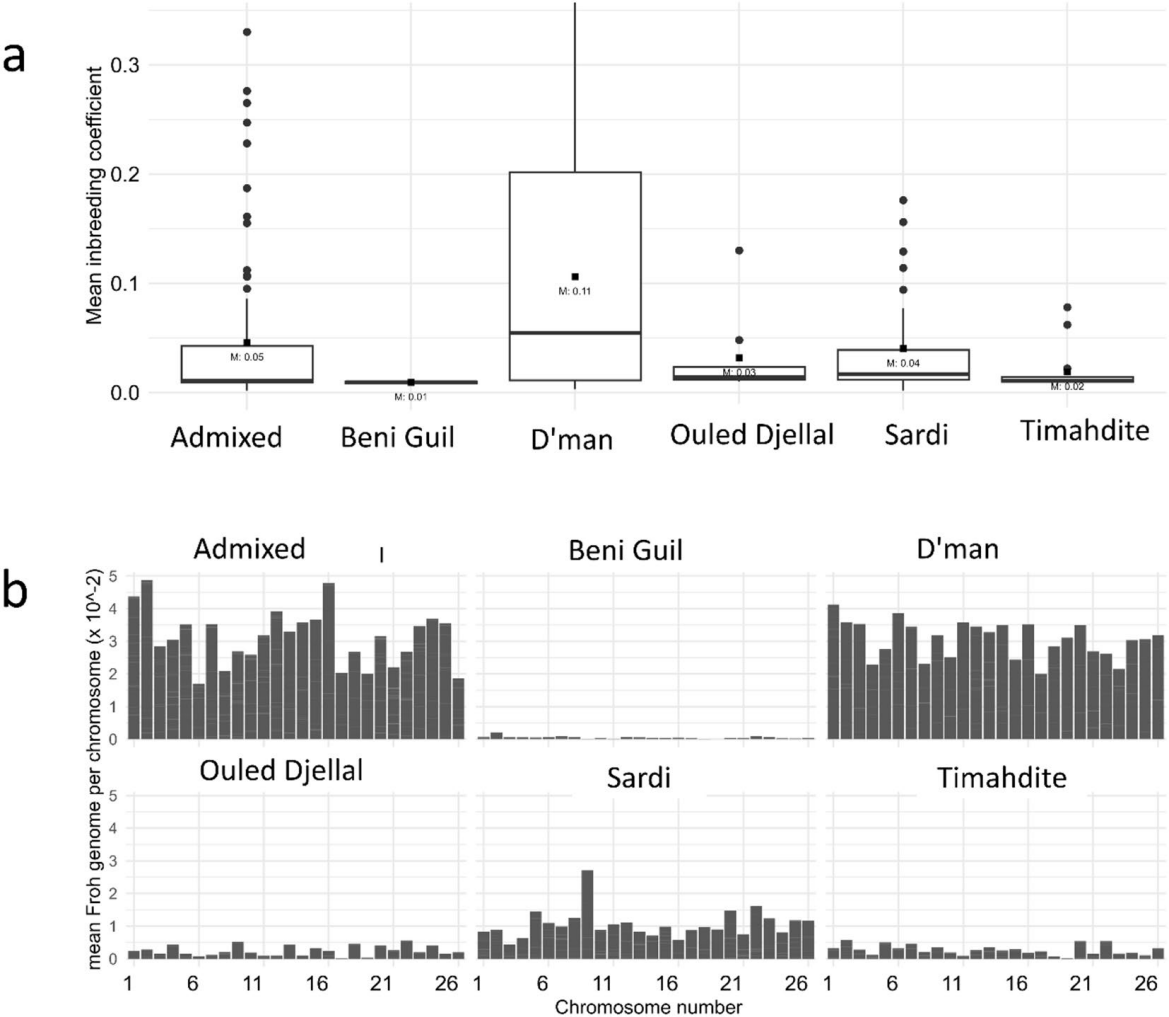
Genomic inbreeding coefficients based on ROH. (**a**): distribution of F_ROH_; (**b)**: breed-specific mean F_ROH_ per chromosome.

Across the 159 samples analyzed, the average ROH length was determined to be 1.83 Mb. Among the breeds studied, D’man displayed the highest frequency of ROH, whereas Beni Guil showed the lowest (Table 1). In terms of ROH segment frequency, the 1–6 Mb segments were most prevalent across all breeds, with the 6–12 Mb segments being significantly less common. Although both the Admixed and D’man breeds had a high frequency of short ROH segments, they were the only breeds that displayed long segments ranging from 24–48 mb, with each breed having just one such segment. The anticipated longest segments of over 48 Mb were absent in all breeds (Table 1). Chromosomal analysis revealed that chromosomes 1 and 2 harbored the highest frequency of SNPs within ROHs, while chromosomes 25 and 26 had the least (Fig. 3a). The Admixed and D’man breeds exhibited the highest frequencies of SNPs within ROHs, in contrast to Beni Guil, which had the lowest (Fig. 3b). The mean inbreeding coefficients obtained in all breeds were generally moderate (mean F_ROH_ = 0.051 ± 0.077, mean F_HOM_ = 0.043 ± 0.079 and F_GRM_ = 0.040 ± 0.080). The F_ROH_ values ranged between 0.106 ± 0.110 (D’man) and 0.009 ± 002 (Beni Guil), F_HOM_ ranged between 0.098 ± 0.113 and 0.001 ± 0.001 while F_GRM_ ranged between 0.002 ± 0.004 and 0.097 ± 0.114 -same breeds. (Table 1 and Fig. 4a). All the three inbreedings coefficients were significantly (*p* < 0.05) highly correlated with each other (F_ROH_-F_HOM_; 0.997, FGRM-_FROH_;0.999, F_GRM_-F_HOM_;0.999). When examining inbreeding levels per chromosome across breeds, the Admixed and D’man breeds demonstrated higher inbreeding levels per chromosome compared to others. Generally, the inbreeding levels per breed mirrored the ROH patterns previously described (Fig. 4b). To determine the potential timeframe in which genetic homogeneity may have originated, two distinct programs for determining effective population size were used and their outcomes were subsequently compared. Supplementary Fig. 2 displays the Ne for every generation, ranging from the most distant to the most recent generation. The SNEP results revealed a decrease in effective population size across all breeds, with a sudden fall occurring between the 350th and 250th generations up to the most recent generation. Nevertheless, the GONE program’s findings revealed varying Ne throughout generations, with all breeds experiencing various bottlenecks. Importantly, the presence of several crossings might serve as a possible signal of genetic admixing.

**Table 1:** Summary of ROH segment counts, mean lengths, and genomic inbreeding coefficients for each breed.

Breed	Mean length (mb ± sd)	F_ROH_ (Mean ± sd)	F_HOM_ (Mean ± sd)		ROH segments				
				F_GRM_ (Mean ± sd)	0–6	6–12	12–24	24–48	> 48
Beni Guil	0.726 ± 0.29	0.009 ± 002	0.001 ± 0.001	0.002 ± 0.004	191	–	–	–	–
D’man	2.280 ± 2.49	0.106 ± 0.110	0.098 ± 0.113	0.097 ± 0.114	3171	215	34	1	–
Ouled Djellal	1.450 ± 1.83	0.032 ± 0.042	0.021 ± 0.041	0.018 ± 0.042	412	16	1	–	–
Sardi	1.677 ± 1.95	0.040 ± 0.054	0.030 ± 0.051	0.026 ± 0.054	1521	62	6	–	–
Timahdite	1.111 ± 1.38	0.019 ± 0.027	0.009 ± 0.019	0.008 ± 0.02	660	5	2	–	–
Admixed	1.733 ± 2.07	0.046 ± 0.074	0.038 ± 0.075	0.035 ± 0.076	4452	186	23	1	–
Mean	1.83 ± 2.16	0.051 ± 0.077	0.043 ± 0.079	0.040 ± 0.080

### ROH islands and gene annotation

ROH islands were defined as genomic regions showing homozygosity in at least 80% of the animals within a specific breed under study. This threshold was initially applied to all 159 animals, leading to the identification of ROH islands only in the Beni Guil breed. To mitigate any potential bias, we applied this threshold to each breed rather than all at once. This adjustment revealed multiple ROH islands across different breeds, with a varying count of SNPs (Table 2). With the exception of Ouled Djellal, which had ROH islands on chromosomes 1 and 5, with the greatest SNP count inside these islands, all other breeds demonstrated ROH islands solely on chromosome 5. Notably, the Timahdite breed did not present any SNPs under this criteria.

**Table 2:** Runs of homozygosity island per breed and genes.

Group	Start SNP	End SNP	Chr	nSNP	From	To	Gene(s)
Beni Guil	ss1173443188	ss1173444258	5	294	107,331,924	107,481,431	*TRNAW-CCA,HS3ST1*
D’man	ss1173442553	ss1173444258	5	305	107,264,142	107,481,431	
Sardi	ss1173443195	ss1173447216	5	788	107,332,067	107,901,446	
Timahdite٭	ss1173443195	ss1173444258	5	294	107,332,067	107,481,431	
Admixed	ss1173442239	ss1173445646	5	558	107,242,889	107,735,313	
Ouled Djellal	ss1173442542	ss1173445721	5	331	107,242,941	107,736,424	
Ouled Djellal	ss1149786736	ss1149804843	1	4791	131,139,367	131,807,430	*ATP5PF, GABPA, JAM2, MRPL39*

٭The islands were defined using quantile.

Thus, to reduce bias in identifying runs of homozygosity (ROH) islands, we adopted the quantile method for their determination of ROH Islands in Timahtide. SNPs within the highest 99.99% frequency were categorized as key SNPs of interest.

Further, to enhance the reliability of the results (genomic regions identified) by the ROH approach, the integrated haplotype score (iHS) method, implemented in the Rehh package in R^21^ within R software, was used to scan the chromosomes/genomic regions that had the largest number of ROH islands. And in this case, only Chr1 and 5 were scanned using this method. “Gene-hunting” and annotation was conducted by manual searches in two complementary databases- NCBI (https://www.ncbi.nlm.nih.gov) and ensemble (https://www.ensembl.org). Gene annotation was done using ARS-UI_Ramb_v3.0 as reference genome. Annotation and gene hunting was done in phases, first phase used ROH islands, and it centered on all identified Islands on Chr5, spanning the genomic regions from the lowest SNP site at 107.3 Mb to the highest SNP site at 107.9 Mb. The second phase focused on the Ouled Djellal breed, specifically annotating Islands found on Chr1, covering a genomic range from 131.1 Mb to 131.8 Mb. Third phase involved scanning the integrated haplotype homozygosity scores (iHS) and delineating regions with outlier SNPs, christened regions of interest (RoI). RoIs were identified by scanning through the genome in sliding windows (window size = 1 mb—similar to the one used in idendifying ROH), and regions that fullifilled the following criteria were selected: “minimum number of markers = 4, minimum number of extremal markers = 4, minimum percentage of extremal markers among the markers = 0 and a log.p value threshold = 3”. The genes identified under ROH islands are detailed in Table 2, while those identified by iHS method are shown in Suppplementary Tables S2 and S3. All the ROH Islands on Chr5 were found to be similar across all breeds, resulting in the identification of two genes: *TRNAW-CCA* and *HS3ST1*. However, the breed Ouled Djellal had its ROH Islands on both Chr1 and Chr5. These ROH Isands on Chr1 revealed four genes: *ATP5PF, GABPA, JAM2*, and *MRPL39*. The results of ROH Islands were consistent with the iHS results, that is, RoI on Chr5 were similar across all breeds. However, the RoI on Chr1 varied among breeds, yielding different genes with Admixed having 52, Beni Guil having 16, D’man having 30, Ouled Djellal having 30, Sardi having 50, and Timahdite having 43 genes. All genes identified in Chr1 were unique to each breed, with only 16 genes being common to all breeds. Among them, all the four genes detected in ROH Islands were also detected by iHS method. Only common genes identified in all breeds per chromosome were further analyszed dowmstream to understand their biological functions. As a result, Metascape (https://metascape.org/)^22^ was used for gene ontology term enrichment analysis, whereas GenMania (https://genemania.org/)^23^ was used to construct a gene–gene interaction network. Furthermore, description of gene functions was enhanced by a comprehensive analysis of the available research articles.

The genes found on Chr1 are mostly associated with “response to stimulus”, “localization”, and “metabolic processes”, (Supplimentary Fig. S4a). Additionally, the gene interaction analysis revealed that *SOD1, SLAMF9, RTP4, CLDN1* and *PRKAA2* exhibit strong connectivity suggesting their vital role in response to stimuli (Supplimentary Fig. S4b). The study revealed that most genes located on Chr5 in sheep are significantly associated with important biological processes, such as the “immune system process”, “regulation of biological process”, and “positive regulation of biological process” (Supplementary Fig. S5a). The gene–gene interaction network (Supplementary Fig. S5b) indicates a substantial connection between *SLC39A1, IL23A, CAST, IL5, IL13*, and *IL4,* suggesting their vital function in the survival of these breeds.

## Discussion

This study involved the analysis of whole genome sequencing from 159 sheep breeds that were sampled from various geographical regions of Morocco. As per the guidelines provided by FAO^24^, the number of samples recommended for genetic characterization studies depends on the density of the markers. It is recommended to have a minimum of 15 samples for higher density markers and 20 samples for medium density markers in order to obtain reliable results. However, Ouchene-Khelifi et al.^14^ used at most 22 samples and atleast 8 samples per breed in to study the “genetic homogeneity of North African goats” using 50 K SNP data. Since our study used whole genome sequencing, we are confident that the results obtained here are reliable and provides a basis of understanding the insights into the genomic homogeneity of Moroccan sheep breeds.

Previous research has consistently highlighted the genetic homogeneity among native small ruminants in North African countries, including Morocco^11,16,17,25^. This study aimed to delve deeper into the characteristics of this homogeneity in sheep through the lens of runs of homozygosity (ROH). Our findings affirm the genetic homogeneity of Moroccan indigenous sheep breeds, as consistently shown in Figs. 1 and 2 alongside F_ST_ values (Supplimentary Table S1), supporting previous documentation and observations. Figure 1a,b demonstrate that there were no evident clusters seen whether based on breed or geographical origin. PC1, PC2, and PC3 account for just 16% of the genetic diversity seen between populations, suggesting a weak population structure. Although the D’man and Sardi breeds showed some divergence along PC1, the Ouled Djellal breed, interestingly positioned at the center, suggested its pivotal role in fostering genetic similarity across these breeds. Historical accounts, as discussed by Belabdi et al.^25^, support the influence of the Ouled Djellal breed on the observed genetic structure. The authors highlighted that the unsupervised crossbreeding of other native breeds with Ouled Djellal could have caused the genetic admixture. Similar genetic uniformity has been reported in North African goats^14,16^, reflecting the shared domestication and migration histories of sheep and goats, hence the observed homogeneity in both species is unsurprising.

Analyzing the ROH, we observed varying segment lengths across breeds, with a predominance of shorter segments (1–6 mb) and a notable scarcity of longer segments (24–28 mb) in only two breeds, while segments longer than 48mb were absent in all. Notably, the D’Man breed exhibited the highest genomic inbreeding based on ROH coefficient (F_ROH_ = 10%) among the breeds studied, with a significant presence of ROH segments within the 6–12 mb and 12–24 mb ranges, indicating a degree of inbreeding higher than initially anticipated. Noteworthy to mention that the Admixed breed, despite its hypothetical genetic mixture, exhibited high ROH compared to standardized breeds challenging conventional expectations. This could theoretically result from bottleneck effects, selection pressures, or sampling biases. Sampling biase may be the possible cause of large number of ROHs in Admixed populations since the sample size of the Admixed population was relatively higher than the other breeds which might have introduced another layer of complexity in analyzing and comparing genetic structures. No significant variations were observed in genomic inbreeding coefficients (F_ROH_, F_HOM_ and F_GRM_) in all breeds and F_ROH_ across chromosomes within individual breeds. All the calculated inbreeding coefficnents were significantly and highly correlated (r > 0.9). The significant high correlation between molecular inbreeding values might be attributed to the fact that the greater amount of inbreeding captured was associated to ROH^26^, and it has also been shown that as *Ne* decrease, the similarity between molecular inbreeding coefficients increase^27^. The ROH segments profile (deficiency of longer ROH segments) suggests that significant genetic homogenization events among these breeds occurred approximately 250–350 generations ago, translating to roughly about 750 to 1050 years ago, assuming a sheep generation interval of three years. Indeed, the analysis of ROH segment profiles, together with the examination of *Ne* trends across generations using GONE and SNEP programs provides evidence that genetic homogeneity happened between 250 and 350 generations back. For instance, Supplementary Fig. S2 reveals that Ouled Djellal, Sardi, and Timahdite saw an increase in their *Ne* from around 300–250 generations ago. These increases were marked by peaks and flactutations, with many trend lines intersecting each other, possibly indicating genetic intermixing. Admixed population had a continuously stable pattern, with a notable increase in genetic exchange occurring around from 300 generations ago. This further substantiates the existence of gene exchange at that particular time period. Similar conclusions may be drawn from Supplementary Fig. S3, which demonstrates that all populations underwent a progressive decrease in *Ne* until around 300 to 250 generations ago which was followed by a rapid decline. The findings align with the chronological sequence of historical events and policies under the French rule, as emphasized by Belabdi et al.^25^, which might be the potential reason for the genetic homogeneity of small ruminants in North Africa.

To discern potential selection signatures within homozygous regions, we delineated ROH Islands. Notably, the Ouled Djellal breed, originally from Algeria, was distinguished by having unique ROH islands on Chr1, contrasting with all other breeds which presented their Islands on Chr5. The inclusion of the Ouled Djellal breed among predominantly Moroccan breeds, emphasizes the transboundary nature of genetic influences and the importance of considering geographical and historical contexts in understanding genetic diversity and uniformity in regional livestock populations. This observation led us to hypothesize that Chr5 might harbor critical genes relevant to domestication, evolutionary processes, and/or adaptability while Chr1 might be the chromosome contributing to the genetic diversity between breeds. A complementary haplotype-based method (iHS) was used to scan for signatures of selection on both Chr1 and Chr5 in all breeds.

Targeted gene searches were performed manually in both ensemble (https://www.ensembl.org/) and NCBI databases (https://www.ncbi.nlm.nih.gov/).

Within the ROH islands spanning 107.3 M to 107.9 M on Chr5, we identified two genes: the transfer RNA tryptophan (anticodon *CCA; TRNAW-CCA)* and Heparan sulfate glucosamine 3-O-sulfotransferase 1 (*HS3ST1*) for all breeds while iHS method yielded 16 genes (same genes for all breeds) among them were genes identified under ROH Island. Gene Ontology term enrichment performed in Metascape (https://metascape.org/) revealed that immunity system and regulation of biological processes are the major pathways that were over-represented by all genes found by both ROH islands and iHS methods (Supplementary Fig. S5). Efficient response to immunity is one of the most important traits of adaptation to environmental circumstances^28–30^, whereas efficient control of biological processes guarantees that physiological activities are kept within ideal parameters, which is crucial for the survival of animals in challenging environments as their misregulation can lead to genome instability and disease^31,32^. Gene interaction netword as developed using GeneMANIA (https://genemania.org/) showed that genes *SLC39A1, IL23A, CAST, IL5, IL13*, and *IL4* are highly connected suggesting their crucial role in immune response and adaptation. Interleukins family genes have highly been linked to immune response^28,33^ while *SLC39A1* gene apart from being responsible for immunity^34^, it is also linked with maintaining of homeostasis^35^ which is also a crucial biological function for adaptation. We hypothesize that genes identified under ROH Island on Chr5 are conserved in all populations and that Chr5 is more homogeneous than other chromosomes. Only genes identified under ROH Island are discussed below.

*TRNAW-CCA* has been associated with body weight in Merino sheep^36,37^ and with tail fatness in sheep adapted to semi-arid environments^38^. Additionally, it has been identified as a gene coding for proteins influential in plasma urea concentrations^39^. *HS3ST1* gene, on the other hand, has been linked to feed efficiency through transcriptome analysis^40,41^ and is suggested to play a role in immunity across species as well^42^.

The Ouled Djellal breed’s ROH islands contained genes such as *ATP5PF, GABPA, JAM2*, and *MRPL39* on Chr1. However, the iHS method produced different numbers of genes in different populations. Notably, the genes found on ROH Island in Oulled Djellal were also among the genes identified by the iHS method, with just 16 genes in common. The gene ontology term enrichment analysis of the common genes showed that the most over-represented biological function was the “response to stimuli”. Response to stimuli is a complex process that encompasses numerous chemical and signaling channels pathways. Genetic diversity has a vital role in altering the expression of genes in response to stimuli. This variation can influence the initiation of certain genes and the formation of intricate molecular networks in response to external stimuli^43^. The studies conducted by Liu et al.^44^ and Lee et al.^45^ highlight the significance of certain stimuli, such as hormones, stressors, darkness, and mechanostimulation, in initiating the binding of regulatory proteins to genes that respond to stimuli. These findings indicate that various stimuli might cause specific genetic reactions, resulting in changes in the control of gene expression and subsequent transcriptional responses. Gene network analysis revealed that genes *SOD1, SLAMF9, RTP4, CLDN1,*and *PRKAA2*, exhibit strong connectivity, suggesting their crucial functions within the response to stimuli. *SOD1* gene for instance is linked to response to exidative stress, adaptation to plateau and also response to hypoxia^46–48^. The *SLAMF9* gene belongs to the Signaling Lymphocyte Activation Molecule Family, and it has been extensively researched in relation to several aspects of sheep adaptability. The study conducted by Zeng et al.^49^ emphasized the function of *SLAMF9* in inhibiting liver inflammation by reducing the expression of *TLR4* on macrophages. This suggests that *SLAMF9* is involved in immune responses and inflammatory processes. It is evident that genetic variability among these breeds is significantly contributed by Chr1. The genes mapped under ROH Islands on Chr1 are discussed below.

ATP5PF is known for its involvement in energy metabolism^50^, *GABPA* has been related to milk production traits^51^, *JAM2* has been implicated in immunity to gastrointestinal parasites^52^, and *MRPL39* is associated with mitochondrial function and aerobic metabolism^53^. This compilation of genes within ROH islands underscores their potential importance in the adaptation and physiological characteristics of these sheep breeds.

This research delved into the distribution of runs of homozygosity (ROH) across the genomes of five indigenous sheep breeds in Morocco, uncovering a significant genomic uniformity among them. Our analysis indicates that this homogeneity stems from inbreeding events occurring between 250 to 350 generations ago, rather than recent breeding practices. ROH islands, or genomic hotspots, emerge through natural selection and can illuminate regions under selective pressure. Except for the Ouled Djellal breed, which displayed unique hotspots on chromosome 1, the detected ROH hotspots across the other breeds were predominantly located on chromosome 5. This consistent localization suggests that chromosome 5 may harbor essential genes crucial for adaptation to arid environments. Notably, study showed that Chr5 is highly homogenous and both ROH Island and iHS methods identified same genes in all breeds under study. The most important biological function of the identified genes on Chr5 was responsible for immunity, a vital adaptation trait in the face of climate change. On the contrary, genomic regions on Chr1 varied between breeds suggesting that Chr1 contributes to the genetic variability seen between breeds. The most over-represented biological function by genes identified in Chr1 was response to stimuli which is also a crucial adaptation trait in the face of climate change. This research not only contributes to the understanding of ROH distribution across breeds but helps design and implement native sheep breeding and conservation strategies in Morocco. Future research, incorporating a broader sample size and utilizing the pangenome for reference, is recommended to further elucidate these breeds’ genomic landscapes and adaptive mechanisms.

## Methods

### Sample description and data management

**Figure 5:**
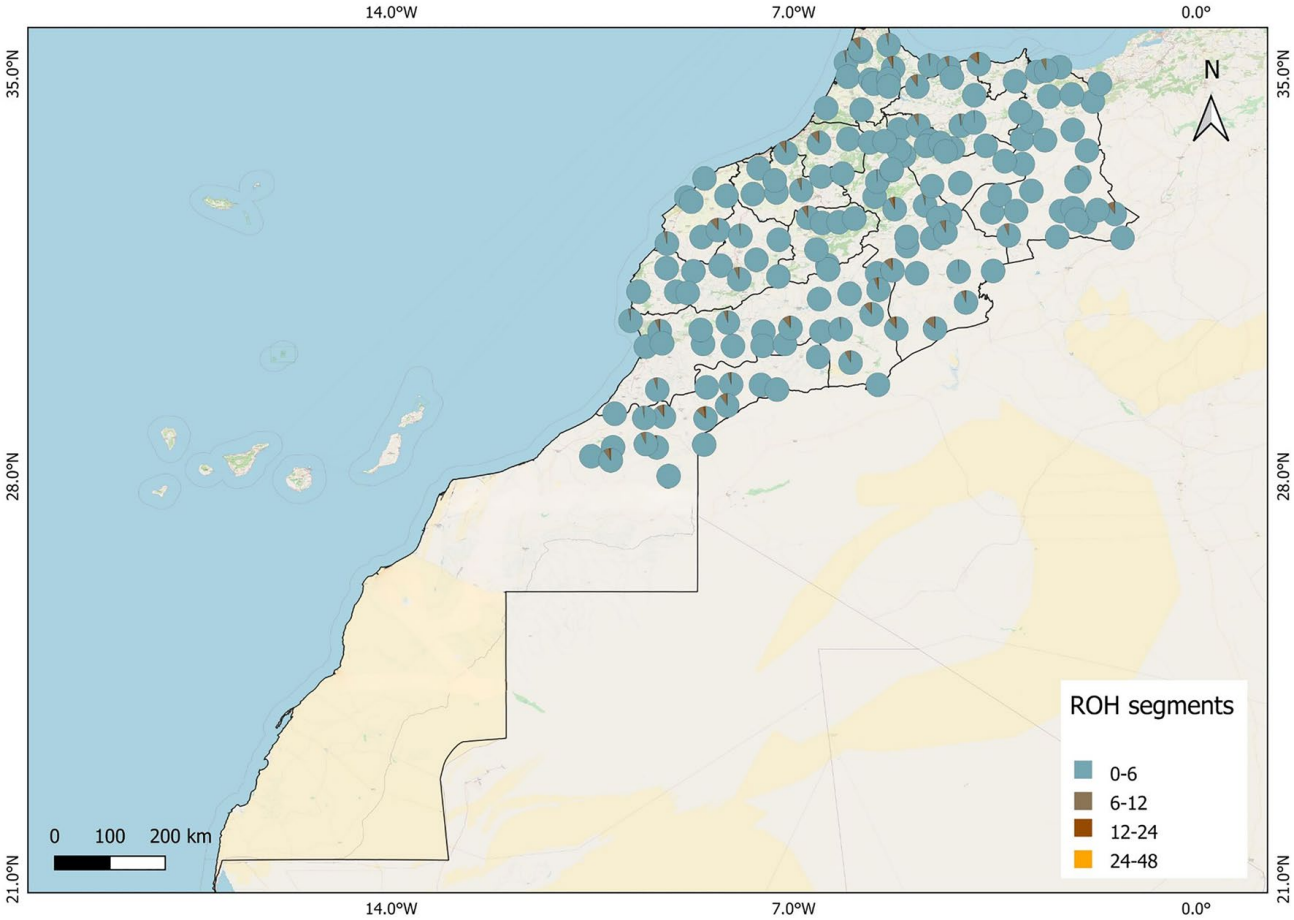
Distribution of the studied breeds and runs of homozygosity segments across Morocco.

This study analyzes whole genome sequence data from 159 sheep samples collected from Morocco, with their distribution illustrated in Fig. 5.It was developed using QBIS.

The breeds examined include Admixed (n = 72), Beni Guil (n = 6), D’man (n = 30), Ouled Djellal (n = 8), Sardi (n = 27), and Timahdite (n = 16). A significant portion of the samples for this research were previously analyzed in Ouhrouch et al.^11^. The methods for sample collection and processing are detailed in Ouhrouch et al.^11^. For identification purposes, samples named after specific breeds were sourced from individuals exhibiting characteristic physical features of those breeds. The Admixed breeds in Morocco are identified as breeds that have adapted locally without displaying the phenotypic features of any known standard breeds, believed to be a product of interbreeding among various indigenous breeds.

The processing of whole genome sequence data was carried out using PLINK 1.9, focusing exclusively on Single Nucleotide Polymorphisms (SNPs) and excluding insertions and deletions (indels) from the analysis. To ensure a high-quality dataset for subsequent investigation, we applied several filtering criteria. We began by discarding variants located on unassigned and sex chromosomes. We also excluded SNPs with a call rate of less than 90% within any given breed, as well as those SNPs with a minor allele frequency (MAF) less than 0.05. This filtering process led to the removal of 36,012 variants failing to meet the MAF threshold and an additional 6,382 variants due to missing genotype information. We also exclude any individual samples based on close familial relationships, such as parent–offspring and full-sibling pairs. After applying these criteria, All samples and 19.7 million SNPs passed the filtres for downstream analysis.

### Population structure

To explore the genetic relationships and spatial arrangements among different sheep breeds, we executed an unsupervised principal component analysis (PCA) on all the breeds, including only the variants that successfully underwent the quality control process^54^. Despite the potential effects of data imbalance on PCA outcomes, we believe that leveraging whole genome sequence data effectively captures significant genetic variations across these breeds. The PCA analysis was carried out using PLINK 1.9, applying the --pca flag. The subsequent PCA plots were generated utilizing the tidyverse package^55^, in R software^56^ which integrates tools for both data visualization and manipulation. The level of differiantiation between breeds was determined by computing F_ST_ using StAMPP package^57^ implemented in R^56^. Neighnour network was developed by split tree (https://mybiosoftware.com/splitstree-compute-phylogenetic-networks.html)^58^ while determination of co-ancestry membership and admixture level was performed by ADMIXTURE 1.3 software (https://dalexander.github.io/admixture/)^59^ .

### Detection of runs of homozygosity and inbreeding

Following the methodology outlined by Meyermans et al.^19^ for identifying runs of homozygosity (ROH), we employed PLINK 1.9 ( https://www.cog-genomics.org/plink/)^60^ with specific configurations, opting out of linkage disequilibrium (LD) pruning. Our approach utilized a sliding window method for ROH detection, employing the -homozyg flag. We set the SNP calling threshold at 0.05, required a minimum of 50 homozygous SNPs within each window, allowed up to 3 heterozygous SNPs, and established a minimum SNP density of 10 SNPs per kb. The maximum permissible gap for homozygosity was 1 mb, with a maximum missingness threshold of 1. To thoroughly assess the lengths of ROHs, we analyzed them at three scales: 100, 500, and 1000 kb, to encompass both short and long ROHs effectively. ROHs were categorized into five length segments: 0–6, 6–12, 12–24, 24–28, and > 48 bp. For additional analyses, such as pinpointing SNPs within ROHs, identifying high-frequency SNPs in each breed, and calculating genome-based inbreeding coefficients (Froh), we utilized the deterctRUNs package^61^. Three inbreeding coefficients, F_ROH_ and F_HOM_, and F_GRM_ were computed and compared. F_ROH_ quantifies the level of autozygosity, which denotes the presence of DNA segments that are homozygous because they are inherited identically from both parents. F_HOM_, on the other hand, measures the overall homozygosity of a population or individual by evaluating the frequency of individuals with two identical alleles at a specific gene locus throughout the genome^62^ while FGRM measures inbreeding using the genomic relationship matrix, considering overall genetic relatedness^26,63^. F_ROH_ was calculated using detectRun package^61^ implemented in R software^56^ whereas the rest were calculated using GCTA (Genome-wide Complex Trait Analysis) program^64,65^. The correlation between inbreeding coefficients was calculated using R software^56^.The identification of ROH islands was carried out using the detectRUNS package implemented in R software (https://www.r-project.org/)^56^, adhering to the criteria that these islands are defined as genomic regions where a certain percentage of individuals within a breed possess SNPs in ROH. Our approach involved a two-phase process. Initially, we examined all samples for the presence of ROH islands, applying an 80% threshold to identify common regions across the sample set. The subsequent phase focused on pinpointing breed-specific ROH islands.

### Supplementary Information

Supplementary Information.

## Data Availability

The data supporting this article can be freely and openly accessed https://ftp.ebi.ac.uk/pub/databases/nextgen/ovis/variants/genus_snps/.
