# Supplementary material for: Insights into the genomic homogeneity of Moroccan indigenous sheep breeds though the lens of runs of homozygosity
Source: Sci Rep. 2024 Jul 17;14:16515. doi: 10.1038/s41598-024-67558-w (PMC11255268; doi:10.1038/s41598-024-67558-w)
Supplement: Supplementary file 1 — Supplementary Information. [file 41598_2024_67558_MOESM1_ESM.docx]

**Supplementary information**

**Table S1:** Fst vaues (p values) per population pair

|  | Admixed | Beni Guil | Sardi | Ouled Djellal | D'man | Timahdite |
| --- | --- | --- | --- | --- | --- | --- |
|  |  |  |  |  |  |  |
| Admixed | 0 |  |  |  |  |  |
| Beni Guil | -0.00122 (1) | 0.00000 |  |  |  |  |
| Sardi | 0.00132 (0) | 0.00175 (0) | 0.00000 |  |  |  |
| Ouled Djellal | 0.00059 (0) | -0.00046 (1) | 0.00290 (0) | 0.00000 |  |  |
| D'man | 0.00144 (0) | -0.00137 (1) | 0.00330 (0) | 0.00114 (0) | 0.00000 |  |
| Timahdite | 0.00080 (0) | 0.00141 (0) | 0.00266 (0) | 0.00299 (0) | 0.00210 (0) | 0 |

The pairwise F_ST_ values between populations revealed very low genetic differentiation. The exceptionally low FST values seen between Admixed and Sardi (0.00132), Ouled Djellal (0.00059), and D'man (0.00144) are significant p < 0.05), while Beni Guil vs. Ouled Djellal (-0.00046) and Beni Guil vs. D'man (-0.00137) are two instances of pairwise comparisons exhibiting significant negative F_ST_ values (p > 0.05. Furthermore, there is a little yet statistically significant genetic variation when comparing Sardi with Ouled Djellal (0.00290), D'man (0.00330), and Timahdite (0.00266).

Possible factors accounting for the absence of genetic divergence including common ancestry, historical gene exchange, or contemporary breeding methods that maintain genetic interconnectedness across populations.

**Table S2**: Genes identified in Chr1 using iHS method

| Timahdite | Ouled Djellal | D’man | BeniGuil | Admixed | Common genes |
| --- | --- | --- | --- | --- | --- |
| UROD | FCER1A | RIT1 | IL12A | UROD | CP |
| IL12A | NCK1 | CP | CP | RIT1 | NCK1 |
| CP | SOD1 | NCK1 | CD1D | CP | KAP7 |
| FCER1A | KAP7 | PRKAA2 | CD1 | CD1D | SOD1 |
| NCK1 | SLAMF9 | SOD1 | KAP7 | CD1 | ATP5PF |
| PRKAA2 | SLCO2A1 | KAP7 | SLAMF9 | FCER1A | SLAMF9 |
| SOD1 | ATP5PF | CYP2J | SLAMF1 | ATP5PF | FCER1A |
| KAP7 | FCGR3A | FCGR2B | ATP5PF | PRKAA2 | PRKAA2 |
| SLAMF9 | FCGR2B | ATP5PF | MRPL39 | SOD1 | RGS4 |
| ATP5PF | RGS4 | RGS4 | AGTR1 | SLAMF9 | ST6GAL1 |
| SLAMF1 | JAM2 | IL12RB2 | MTERF4 | SLCO2A1 | CLDN1 |
| NUDT16 | RABGGTB | RXRG | SPP2 | CYP2J | SST |
| MX2 | ATP5PO | MRPL39 | CD86 | SLAMF1 | RTP4 |
| MX1 | IFNAR1 | RABGGTB | JAM2 | ITLN | ADIPOQ |
| JAM2 | IFNAR2 | RCAN1 | GHSR | FCGR3A | AHSG |
| CRYAA | SPP2 | ATP5PO | ARNT | FCGR2B |  |
| PDXK | UGT1A1 | CYM |  | JAM2 |  |
| GATD3 | CD80 | CD53 |  | RGS4 |  |
| ITGB2 | GSK3B | ATP1A1 |  | IL12RB2 |  |
| MRPL39 | ATP1A1 | HSD3B1 |  | CRYAA |  |
| ATP5PO | FGF12 | FGF12 |  | MRPL39 |  |
| IFNAR1 | CLDN1 | CLDN1 |  | GJA8 |  |
| AGTR1 | SST | SST |  | RABGGTB |  |
| BTG3 | RTP4 | RTP4 |  | RCAN1 |  |
| POU1F1 | ST6GAL1 | ST6GAL1 |  | ATP5PO |  |
| CYM | ADIPOQ | ADIPOQ |  | IFNAR1 |  |
| CD53 | AHSG | AHSG |  | IFNAR2 |  |
| OVGP1 | PRD-SPRRII | GHSR |  | AGTR1 |  |
| ADORA3 | ARL4C | ARL4C |  | BTG3 |  |
| RNF168 |  |  |  | POU1F1 |  |
| HSD3B1 |  |  |  | SPP2 |  |
| FGF12 |  |  |  | UGT1A1 |  |
| YBX1 |  |  |  | CD80 |  |
| CLDN1 |  |  |  | GSK3B |  |
| SST |  |  |  | RRAGC |  |
| RTP4 |  |  |  | CD86 |  |
| ST6GAL1 |  |  |  | PPIE |  |
| ADIPOQ |  |  |  | RNF168 |  |
| FCGR1A |  |  |  | HSD3B1 |  |
| ARNT |  |  |  | FGF12 |  |
| PSMD4 |  |  |  | CLDN1 |  |
| PRD-SPRRII |  |  |  | SST |  |
|  |  |  |  | RTP4 |  |
|  |  |  |  | ST6GAL1 |  |
|  |  |  |  | ADIPOQ |  |
|  |  |  |  | AHSG |  |
|  |  |  |  | FCGR1A |  |
|  |  |  |  | GHSR |  |
|  |  |  |  | ARNT |  |
|  |  |  |  | PSMD4 |  |
|  |  |  |  | ARL4C |  |

**Table S3:** Genes identified in Chr5 using iHS method

| Admixed | Beni Guil | Dman | OuledDjellal | Sardi | Timahdite |
| --- | --- | --- | --- | --- | --- |
| CYP4F21 | CYP4F21 | CYP4F21 | CYP4F21 | CYP4F21 | CYP4F21 |
| SQSTM1 | SQSTM1 | SQSTM1 | SQSTM1 | SQSTM1 | SQSTM1 |
| ELAVL1 | ELAVL1 | ELAVL1 | ELAVL1 | ELAVL1 | ELAVL1 |
| CCL25 | CCL25 | CCL25 | CCL25 | CCL25 | CCL25 |
| TRNAW-CCA | TRNAW-CCA | TRNAW-CCA | TRNAW-CCA | TRNAW-CCA | TRNAW-CCA |
| RACK1 | RACK1 | RACK1 | RACK1 | RACK1 | RACK1 |
| HS3ST1 | HS3ST1 | HS3ST1 | HS3ST1 | HS3ST1 | HS3ST1 |
| ZNF496 | ZNF496 | ZNF496 | ZNF496 | ZNF496 | ZNF496 |
| HAND1 | HAND1 | HAND1 | HAND1 | HAND1 | HAND1 |
| IL12B | IL12B | IL12B | IL12B | IL12B | IL12B |
| SLC39A3 | SLC39A3 | SLC39A3 | SLC39A3 | SLC39A3 | SLC39A3 |
| IL4 | IL4 | IL4 | IL4 | IL4 | IL4 |
| IL13 | IL13 | IL13 | IL13 | IL13 | IL13 |
| IL5 | IL5 | IL5 | IL5 | IL5 | IL5 |
| IRF1 | IRF1 | IRF1 | IRF1 | IRF1 | IRF1 |
| CAST | CAST | CAST | CAST | CAST | CAST |

Same genes were mapped out in all the populations on chromosome 5. Also, all genes mapped in ROH islands were identified here.

**
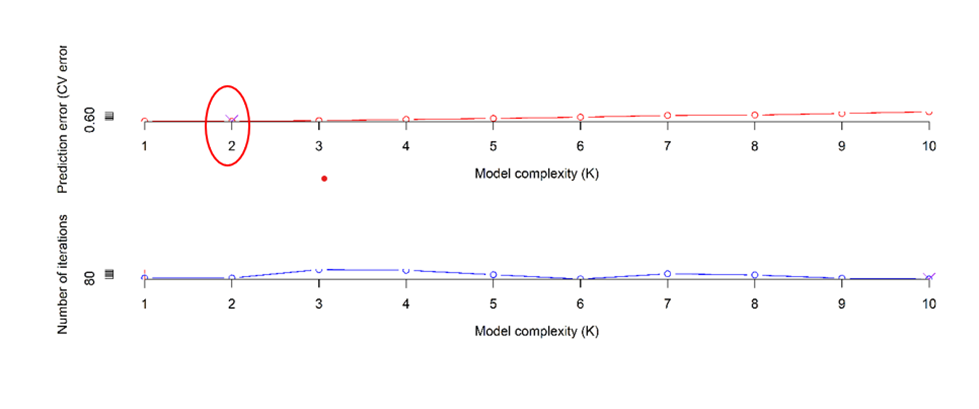
**

**Supplementary Figure S1:** Cross-validation error values (Upper) and number of iterations required to reach convergence (lower) calculated through Admixture software runs for K values ranging from 2 to 10. The red circle indicates the K = 2 value with the lowest CV score.


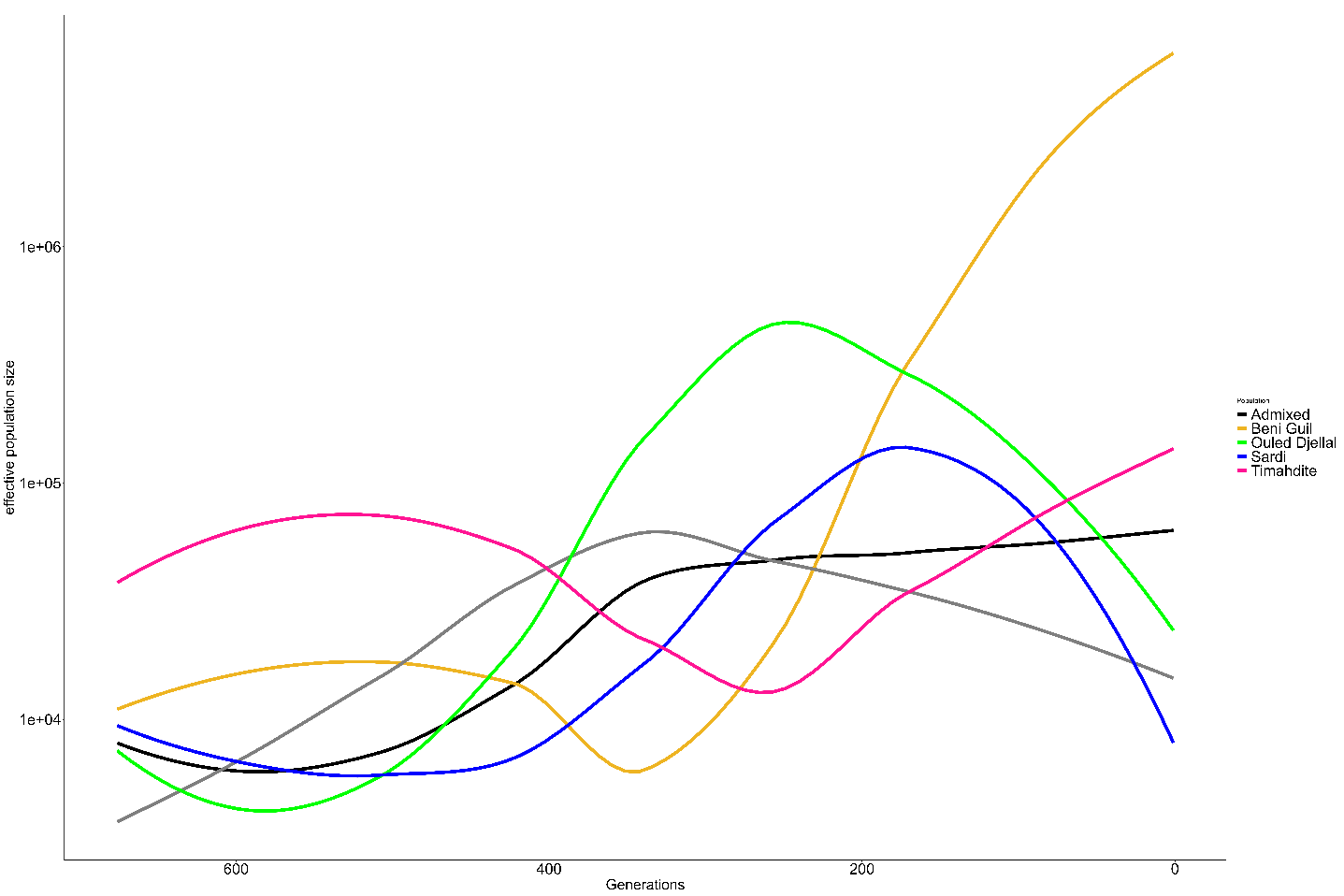


**Supplementary Figure S2**: Effective population size by GONE program


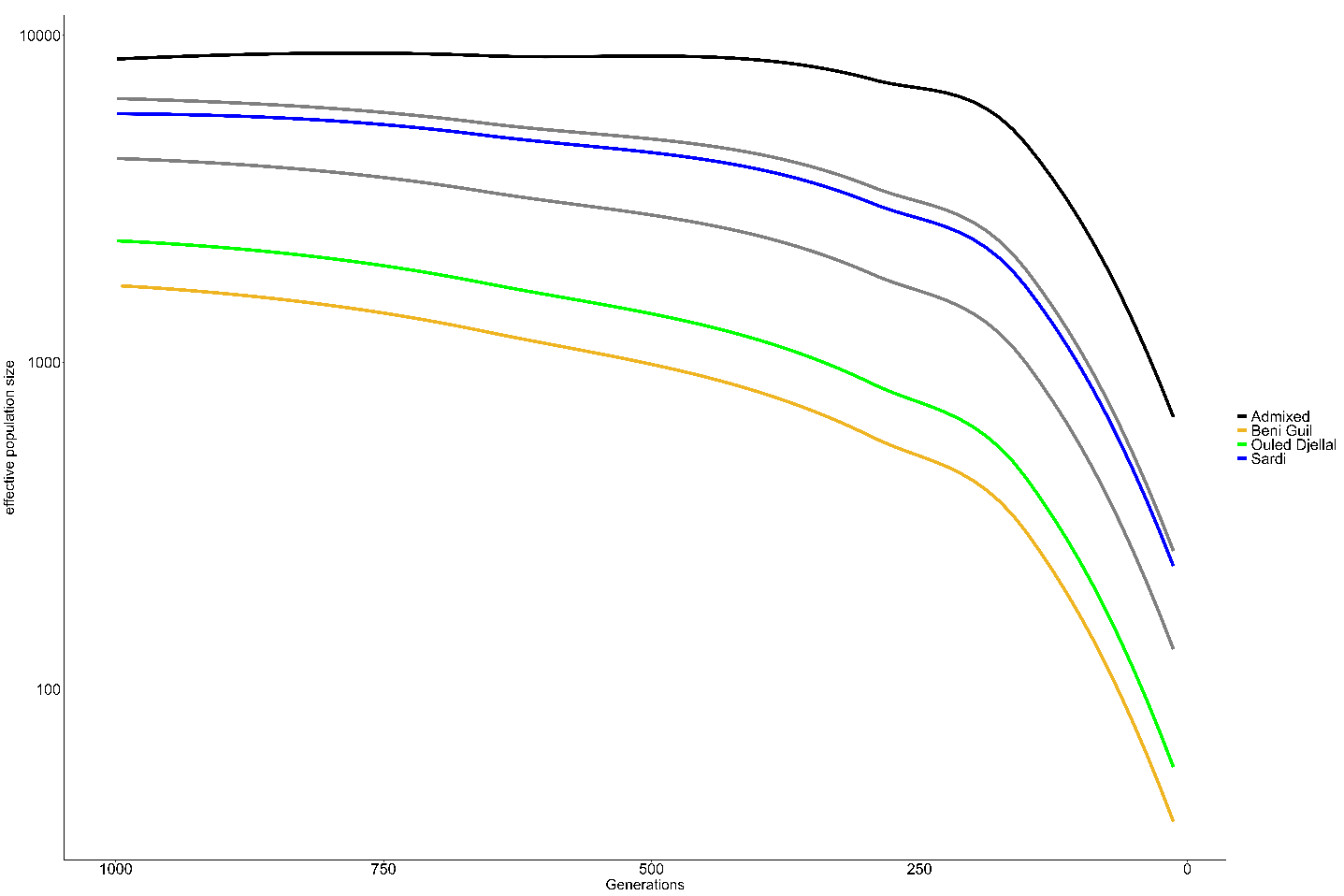


**Supplementary Figure S3**: Effective population size by SNEP program

Examining the effective population size across numerous generations provides valuable information on the genetic history of the populations under study. Both graphs (Supplementary Figures S2 and S3) demonstrate a time of possible genetic homogenization and exchange of genes. The Supplementary Figures S2 demonstrate that groups such as Ouled Djellal, Sardi, and Timahdite have seen significant growth in their effective population size from about 300 to 250 generations ago. These increases are characterized by peaks and fluctuations, indicating the introduction of genetic diversity. Similarly, Admixed group has a consistently steady pattern, but there is a noticeable surge in genetic exchange around 300 generations ago. This provides more evidence for the presence of gene sharing at that specific time period.

The Supplementary Figures S3 support these findings by demonstrating a steady drop in effective population sizes, particularly evident approximately 300 to 250 generations ago. This tendency is consistent with the theory that there is an increase in genetic admixture, which results in a homogeneity impact on the genetic makeup of sheep populations. As a result, the different population “identities” become less clear, leading to a decrease in the effective population numbers.


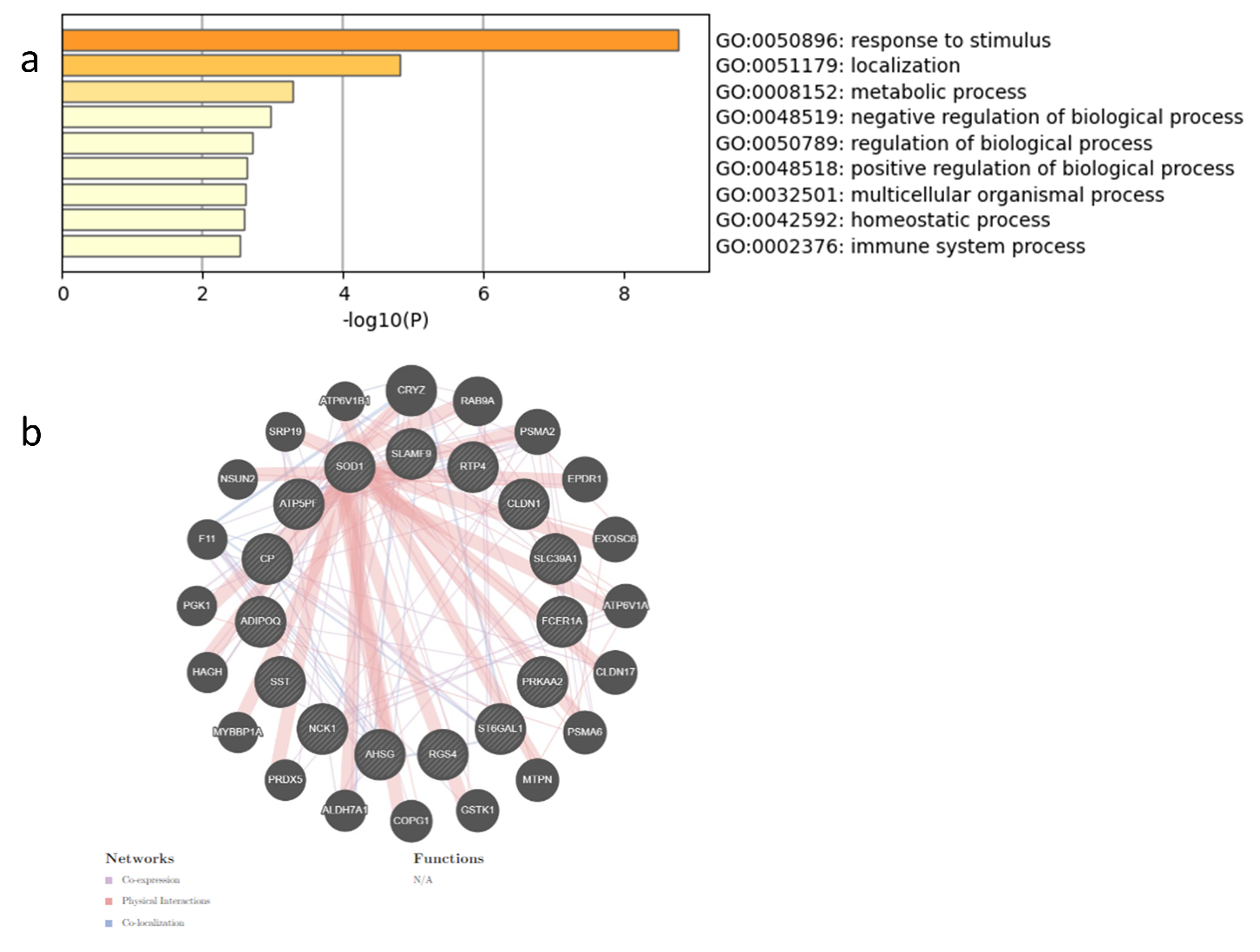


**Supplementary Figure S4**: Gene ontology enrichment terms for common genes found in all the breeds on Chr1 by iHS method.

a; Heatmap for Gene-Ontology enrichment term , b; Gene-gene interaction network

The enrichment analysis indicates that these genes are strongly represented by the top three Gene Ontology (GO) terms: "response to stimulus," "localization," and "metabolic process" (Supplementary Figure S4a). The GO phrase "Response to stimulus" is highly enriched in sheep, suggesting that they play a crucial role in the systems to detect and respond to changes in their environment. Adapting quickly to changes in the desert environment is essential, as circumstances might vary fast, necessitating organisms to promptly modify their physical and behavioral characteristics.

Localization refers to the accurate transportation and placement of chemicals within cells. This function is crucial for preserving cellular balance and optimizing metabolic processes, especially in the challenging conditions of a desert climate. Efficient localization systems guarantee the proper distribution and utilization of resources within the body, particularly in situations where feed and water resources are scarce.

The metabolic process- emphasizing the significance of metabolic efficiency in sheep adapted to arid environments. Metabolic processes involve biochemical activities that transform food into energy, a crucial activity in situations with little food and water resources. Sheep have developed comparable metabolic techniques to efficiently extract energy and avoid waste, allowing them to flourish in the challenging and resource-limited desert environment. This is indicated by the genetic uniformity observed in their metabolic genes. The sheep's enhanced metabolic processes demonstrate its ability to efficiently collect and utilize energy, which is essential for its survival in an environment with limited food supplies.

The network visualization provides a clearer understanding of the genetic relationships that are responsible for these enhanced biological activities (Supplementary Figure S4b). The central genes, including *SOD1, SLAMF9, RTP4, CLDN1* and *PRKAA2*, exhibit strong connectivity, suggesting their crucial functions within the genetic network. *SOD1* plays a vital role in the cellular response to oxidative stress, safeguarding cells against harm resulting from elevated levels of oxidative stress commonly found in desert environments. *SLAMF9* and other immune-related genes, such as *FCER1A*, underscore the significance of a resilient immune system in safeguarding against infections and environmental adversities. *PRKAA2* has a pivotal role in energy metabolism, facilitating effective energy regulation that is vital for survival in environments with limited resources. *CLDN1* plays a crucial role in preserving the integrity of cellular structure and facilitating the movement of ions, which are essential for maintaining the resilience and proper functioning of cells. The discovery of densely interconnected core genes offers novel understanding into the essential genetic elements that propel adaptation, highlighting their critical functions in upholding cellular function and reacting to environmental pressures.

**
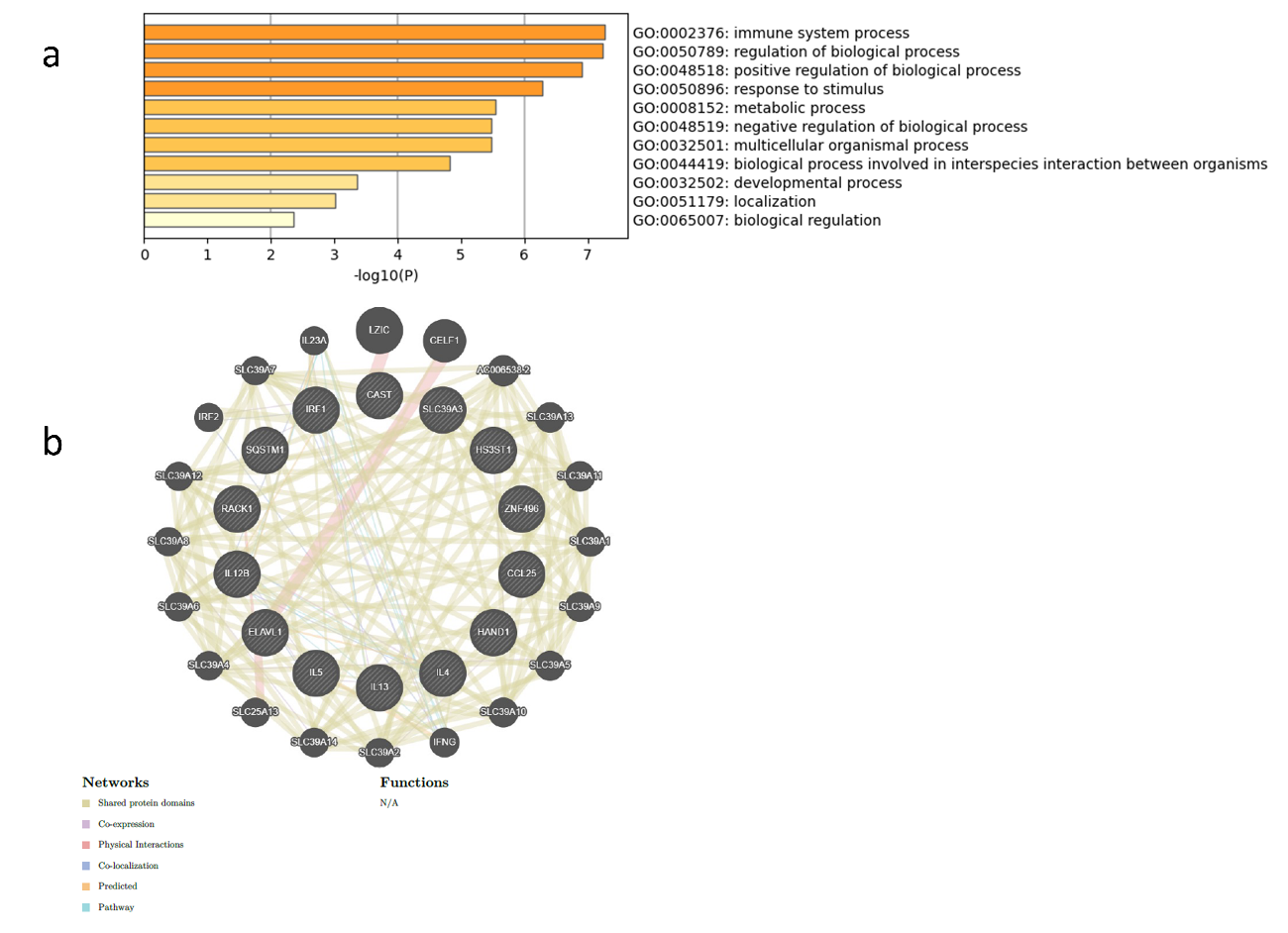
**

**Supplementary Figure S5:** Gene ontology enrichment terms for genes found in all the breeds by iHS method on chromosome 5.

a; Heatmap for Gene-Ontology enrichment term , b; Gene-gene interaction network

NB: All the genes found using ROH Islands are part of the genes identified using iHS method.

The GO enrichment term analysis indicates that the most vital biological processes for the sheep's survival in the desert environment are "immune system process," "regulation of biological process," and "positive regulation of biological process." Efficiently controlling immunological responses is vital for sheep to defend against infections and environmental threats. Efficient control of biological processes guarantees that physiological activities are kept within ideal parameters, which is crucial for the survival of animals in these environments. Positive control of biological processes leads to the enhancement of specific beneficial activities, which ultimately improves the general fitness and adaptability of sheep.

The gene interaction network reveals the genetic relationships that underlie these enhanced biological activities. The genes *SLC39A1, IL23A, CAST, IL5, IL13*, and *IL4* are central in the genetic network, since they are strongly interconnected, suggesting their crucial importance. The network displays common protein domains, co-expression, physical connections, and co-localization among the genes. Genes such as *SLC39A1* have a role in the transportation of zinc ions, which is essential for a range of cellular activities, including immunological function. *IL23A*, *IL5, IL13*, and *IL4* are cytokines that have important functions in immunological responses, suggesting that the strength and effectiveness of the immune system is a crucial adaptation.
